# Corticophobia and adherence to topical corticosteroids in atopic dermatitis treatment in southern Brazil^☆^

**DOI:** 10.1016/j.abd.2023.04.008

**Published:** 2024-02-19

**Authors:** Bruna Ossanai Schoenardie, Gabriela Fortes Escobar, Jéssica Pauli Damke, Gabriel Cardozo Müller, Renan Rangel Bonamigo

**Affiliations:** a Dermatology Service, Hospital de Clínicas de Porto Alegre, Porto Alegre, RS, Brazil; b Programa de Pós-Graduação em Epidemiologia, Faculdade de Medicina, Universidade Federal do Rio Grande do Sul, Porto Alegre, RS, Brazil; c Programa de Pós-Graduação em Ciências Médicas, Universidade do Vale do Taquari, Lajeado, RS, Brazil; d Dermatology Service, Santa Casa de Misericórdia de Porto Alegre, Porto Alegre, RS, Brazil

Dear Editor,

Atopic Dermatitis (AD) is an inflammatory dermatosis with a very high prevalence worldwide. Due to the chronic nature of AD, good adherence to treatment is imperative. Recent studies in developed countries have found a link between corticophobia and poor adherence to treatment in AD. In Brazil, only a few data are available on this subject.^1^

This is a cross-sectional study performed at a University Hospital in Southern Brazil. Subsequent patients of all ages who attended the Outpatient Dermatology Clinic from June 2021 to March 2022 were invited to participate. The inclusion criterion was a diagnosis of AD according to the UK Working Party. The exclusion criterion was never having used Topical Corticosteroids (TCS) before. This study was approved by the hospital's Ethics in Research Committee (number 2021-0091). An informed consent form was provided and signed by either the patient or the caretaker.

All analyses were conducted on R Studio (version 2022.2.2.485), an environment for Statistical computing R (version 4.2.0). Quantitative variables were analyzed in accordance with their distribution symmetry, expressed in either mean and standard deviation or median and interquartile range. In this context, either Student’s T or the Mann-Whitney test for independent samples were used. To analyze the correlation between quality of life and corticophobia scores, Spearman and Kendall methods were used. All analyses were bicaudal and the value of alpha was set at 0.05.

The sample size was calculated to associate adherence to treatment to the degree of corticophobia (utilizing the TOPICOP ‒ Topical Corticosteroid Phobia ‒ questionnaire) using PSS Health version 0.1.5,^2^ based on data by Lee et al.^3^ We set power at 80% and significance level at 5%. Utilizing the effect size of 0.382 found by Lee et al., we estimated a sample size of 75 patients.

TOPICOP is a validated questionnaire that assesses corticophobia.^1^, ^4^ It is composed of 12 questions, divided into two domains: worries and beliefs.^4^ All questions offer four possible answers. Results are presented as percentages (higher scores indicate more corticophobia). For patients <17 years of age, the primary caretaker answered the TOPICOP questionnaire.

We included a total of 75 patients. The mean TOPICOP(t) score was 41.44 (±21.58). Table 1 summarizes patients’ characteristics.

**Table 1:** Summary of patients’ characteristics.

		Children and Adolescents (< 17 years)	Adults (≥ 17 years)	p
n		38	37	
**Age, mean (SD)**		8.79 (5.57)	33.65 (15.57)	
**Sex (%)**	Male	20 (52.6)	13 (35.1)	
**Family history (%)**	Yes	9 (23.7)	14 (37.8)	
**Affected relative (%)**	Father	0 (0.0)	0 (0.0)	
	Mother	2 (22.2)	2 (14.3)	
	Sibling	2 (22.2)	6 (42.9)	
	Cousin	0 (0.0)	1 (7.1)	
	Other	5 (55.6)	5 (35.7)	
**Educational level in years, mean (SD)**		‒	11.11 (2.69)	
**Primary caretaker’s educational level in years, mean (SD)**		10.61 (3.05)	‒	
**Self-reported adherence to TCS (%)**	Not always	15 (39.5)	12 (32.4)	0.693
	Always	23 (60.5)	25 (67.6)	
**Ever used TCS without prescription (%)**	Yes	17 (44.7)	23 (62.2)	0.200
**Ever used TCS for extended period (%)**	Yes	11 (28.9)	20 (54.1)	0.048
**Ever used TCS with increased frequency (%)**	Yes	12 (31.6)	14 (37.8)	0.744
**TOPICOP(t), mean (SD)**		45.18 (23.07)	37.61 (19.51)	0.130
**SCORAD (median [IQR])**		26.68 [18.43‒45.17]	30.01 [14.26‒43.26]	0.916

We found no association between corticophobia and rates of self-reported adherence to treatment (dichotomized in two groups: “always adheres completely” and “does not always adhere”) (p = 0.3797).

Caretakers with higher educational levels presented less corticophobia [TOPICOP(t)]; as seen in Fig. 1 (p = 0.005132). Among the adults, there was no association (Fig. 1; p = 0.7856). Caretakers scored higher than adult patients in TOPICOP(b) (p = 0.02648; Fig. 2). Adults with a higher educational level had better adherence (p = 0.024). DLQI scores did not correlate with corticophobia; however, higher CDLQI scores correlated with higher TOPICOP(t) scores (Fig. 3; p = 0.0086). SCORAD did not correlate with TOPICOP(t) (p = 0.3189).

**Figure 1:**
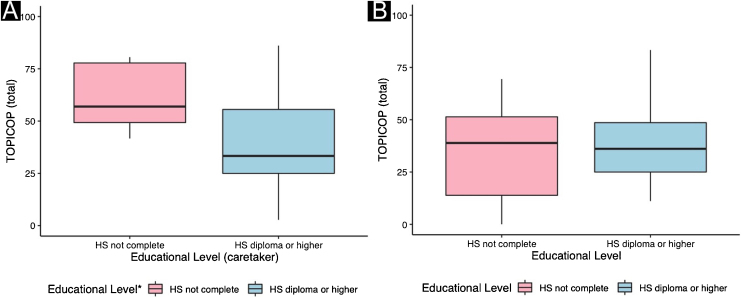
**Correlation between educational level and corticophobia.** (A) Patients < 17 years of age; caretaker’s educational level and TOPICOP(t). (B) Patients ≥ 17 years of age; patient’s educational level and TOPICOP(t). *Caretaker’s educational level; HS, High School.

**Figure 2:**
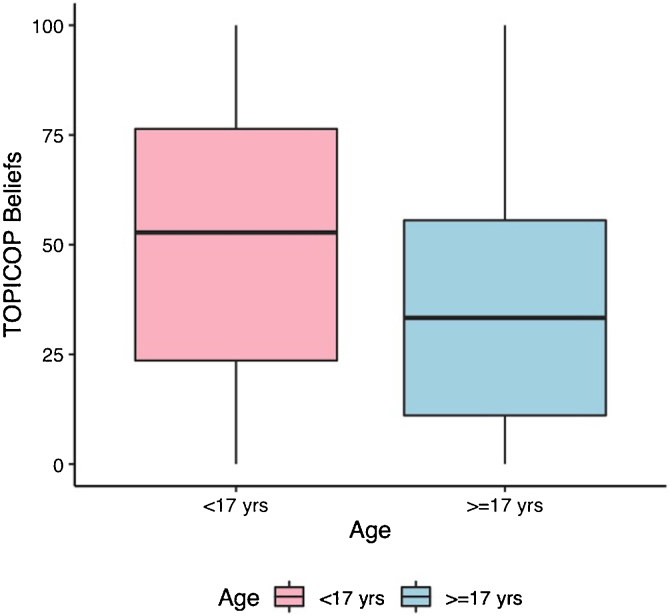
**Comparison of corticophobia between age groups.** Yrs, Years.

**Figure 3:**
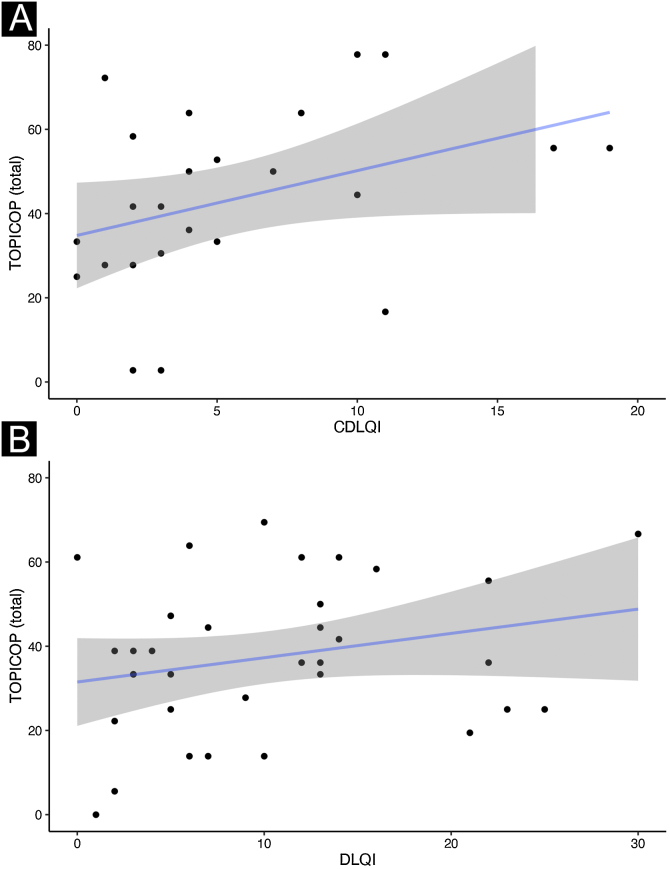
**Correlation between Quality-of-Life indexes and corticophobia.** (A) CDLQI and TOPICOP(t). (B) DLQI and TOPICOP(t).

We assessed sources of information regarding TCS (more than one option was allowed). Only 65.33% (n = 49) responded they got information from a dermatologist. Patients who reported getting information from friends and family (10.66%; n = 8) had less corticophobia than those who did not, in the univariate analysis (p = 0.028). However, our study did not have enough power to perform a multivariable analysis and this information needs to be confirmed with further studies.

TOPICOP(t) scores among caretakers were similar to those previously described.^5^, ^6^ Overall TOPICOP(t) scores were higher than those reported by Stalder et al.^1^ in São Paulo, Brazil (mean = 33.5). However, approximately half of our patients were under 17, which might explain more corticophobia, and the sample size in Stalder et al. was small (n = 48). Additionally, Brazil is a country with substantial social, educational, and cultural differences among its regions and states. Higher TOPICOP(t) scores among caretakers than adult patients indicate that often parents are more concerned about their children’s health than they are about their own. A recent Portuguese study did not find similar results. However, the two studies used different cut-points to separate children/adolescents from adults.^7^ and the populations assessed were distinct (West-European patients compared to patients from a developing country in South America). They also did not assess adherence or quality of life.

We found no association between corticophobia and adherence. Noteworthily, adherence was assessed retrospectively, due to methodological limitations, which might result in recall bias, and was self-reported by the patients, which could generate response bias.

An association between corticophobia and adherence to treatment has been described,^3^ but it is not yet a consensus.^8^ Interestingly, a study by Mueller et al. found that corticophobia measured by the Visual Analogue Scale (VAS) was associated with poorer adherence, however when applying the TOPICOP questionnaire in the same sample it was not.^9^ It has not yet been defined a cut-off point in TOPICOP scores which can be classified as clinically relevant.^5^

We often see patients in Southern Brazil who are not aware an specific formulation contains steroids. Therefore, although corticophobia rates were comparable to those seen in developed countries, it is possible they might not affect adherence to treatment in Brazil to the same degree for this reason.

Differently from ours, previous studies have described an association between educational level and corticophobia.^5^ However, there is a possibility that cultural characteristics might be involved since most studies were performed in Northern Western countries. A Chinese study found no association.^8^

Our study suggests that people who get their information regarding TCS from friends and family might have less corticophobia, which goes in agreement with Song et al.^10^ This is especially relevant in Brazil, where TCS is sold freely at drugstores without the need for a prescription. Over half of our patients had purchased TCS without a prescription in the past. Only 65% responded a dermatologist was one of their main sources of information. This indicates a need to better educate our patients when prescribing treatment for AD. Interestingly, a recent Portuguese study found an inverse association between health literacy and corticophobia.^7^

There appears to be no association between corticophobia and adherence to treatment in Southern Brazil. Additionally, lower educational levels are associated with a higher degree of corticophobia among caretakers of children and adolescents with AD and lower adherence among adults.

## Financial support

This project received funding from *Fundo de Incentivo à Pesquisa e Eventos* (FIPE) of Hospital de Clínicas de Porto Alegre.

G.C. Müller received fees from the World Health Organization (WHO – PAHO) as a consultant.

## Authors' contributions

Bruna Ossanai Schoenardie: Design and planning of the study; data collection, or analysis and interpretation of data; statistical analysis; drafting and editing of the manuscript or critical review of important intellectual content; collection, analysis and interpretation of data critical review of the literature; approval of the final version of the manuscript.

Gabriela Fortes Escobar: Design and planning of the study; data collection, or analysis and interpretation of data; collection, analysis and interpretation of data; effective participation in research orientation; intellectual participation in the propaedeutic and/or therapeutic conduct of the studied cases; critical review of the literature; approval of the final version of the manuscript.

Jéssica Pauli Damke: Data collection, or analysis and interpretation of data; collection, analysis and interpretation of data; approval of the final version of the manuscript.

Gabriel Cardozo Müller: Data collection, or analysis and interpretation of data; statistical analysis; drafting and editing of the manuscript or critical review of important intellectual content; collection, analysis and interpretation of data; approval of the final version of the manuscript.

Renan Rangel Bonamigo: Design and planning of the study; drafting and editing of the manuscript or critical review of important intellectual content; collection, analysis and interpretation of data; effective participation in research orientation; intellectual participation in the propaedeutic and/or therapeutic conduct of the studied cases; critical review of the literature; approval of the final version of the manuscript.

## Conflicts of interest

None declared.

## References

[1] Stalder J.F., Aubert H., Anthoine E., Futamura M., Marcoux D., Morren M.A. (2017). Topical corticosteroid phobia in atopic dermatitis: international feasibility study of the TOPICOP score. Allergy..

[2] Borges RB, Azambuja GS, Mancuso ACB, Leotti VB, Hirakata VN, Camey SA, et al. PSS.Health: power and sample size for health researchers via Shiny. R package version 0.1.5. 2020. Available from: https://CRAN.R-project.org/package=PSS.Health.

[3] Lee J.Y., Her Y., Kim C.W., Kim S.S. (2015). Topical corticosteroid phobia among parents of children with atopic eczema in Korea. Ann Dermatol..

[4] Moret L., Anthoine E., Aubert-Wastiaux H., le Rhun A., Leux C., Mazereeuw-Hautier J. (2013). TOPICOP©: a new scale evaluating topical corticosteroid phobia among atopic dermatitis outpatients and their parents. PLoS One..

[5] Dufresne H., Bataille P., Bellon N., Compain S., Deladrière E., Bekel L. (2020). Risk factors for corticophobia in atopic dermatitis. J Eur Acad Dermatol Venereol..

[6] Saito-Abe M., Futamura M., Yamamoto-Hanada K., Yang L., Suzuki K., Ohya Y. (2019). Topical corticosteroid phobia among caretakers of children with atopic dermatitis: a cross-sectional study using TOPICOP in Japan. Pediatr Dermatol..

[7] Gomes T.F., Kieselova K., Guiote V., Henrique M., Santiago F. (2022). A low level of health literacy is a predictor of corticophobia in atopic dermatitis. An Bras Dermatol..

[8] Hon K.L., Tsang Y.C.K., Pong N.H., Luk D.C.K., Lee V.W., Woo W.M. (2015). Correlations among steroid fear, acceptability, usage frequency, quality of life and disease severity in childhood eczema. J Dermatolog Treat..

[9] Mueller S.M., Itin P., Vogt D.R., Walter M., Lang U., Griffin L.L. (2017). Assessment of “corticophobia” as an indicator of non-adherence to topical corticosteroids: a pilot study. J Dermatolog Treat..

[10] Song S.Y., Jung S.Y., Kim E.Y. (2019). Steroid phobia among general users of topical steroids: a cross-sectional nationwide survey. J Dermatolog Treat..

